# Medication experiences in the treatment of opioid use disorders: Insights from Reddit

**DOI:** 10.1111/add.70022

**Published:** 2025-03-13

**Authors:** Alexandra Almeida, Mike Conway, David J. Grelotti, Amarnath Gupta, David Frank, Annick Bórquez

**Affiliations:** ^1^ Scientific Computing Program Oswaldo Cruz Foundation Rio de Janeiro Brazil; ^2^ Centre for Digital Transformation of Health University of Melbourne Carlton Australia; ^3^ Department of Psychiatry UC San Diego School of Medicine San Diego CA USA; ^4^ San Diego Supercomputer Center University of California San Diego La Jolla CA USA; ^5^ Department of Social and Behavioral Sciences and Center for Drug Use and HIV/HCV Research, School of Global Public Health New York University New York NY USA; ^6^ Division of Infectious Diseases and Global Public Health University of California San Diego San Diego CA USA

**Keywords:** infodemiology, medication experience, MOUD, NLP, opioids, reddit

## Abstract

**Background and Aims:**

Better understanding the challenges faced by patients on medications for opioid use disorder (MOUD), including methadone and buprenorphine, is critical to increasing their use/retention. Social media platforms such as Reddit offer a space for patients to share their experiences with medications. We aimed to identify and characterize challenges faced by patients taking MOUD through analysis of discussions from the r/Methadone and r/suboxone subreddits.

**Design, Setting and Participants:**

Mixed methods study applying natural language processing methods to 37 278 posts from both subreddits from their origin in 2011 until 31 December 2022. Independent topic analyses used Correlated Topic Models to extract the main themes discussed.

**Measurements:**

We labeled, validated and grouped the topics into macro classes and computed topic shares. We interpreted and compared topics across subreddits informed by the patient‐centered medication experience framework.

**Findings:**

We found 27 and 34 challenge‐related topics for the r/Methadone and r/suboxone subreddits, respectively. Topics were grouped into three macro‐topics: (i) healthcare‐related issues, including misunderstandings/confusion around appointments, prescriptions, bottle checks, telehealth technology and health insurance coverage; (ii) medication‐related issues, including withdrawal, cravings, dosage, side effects, mixing with other medications/drugs; and (iii) treatment discontinuation, including tapering protocols. Patients conveyed highly specialized knowledge about dosage and tapering strategies and spoke from experience. Key differences between r/Methadone and r/suboxone were driven by their dispensing requirements (clinic‐based vs. take‐home), with 20.05% vs 14.74% of posts related to healthcare service, primarily for logistic and interpersonal issues with healthcare providers.

**Conclusion:**

People who post on the r/Methadone and r/suboxone subreddits appear to have detailed knowledge of medications for opioid use disorder and want more control over their dosing, effects, side effects and discontinuation. Acknowledging this expertise and establishing stronger patients' partnerships with the healthcare team and system might result in better treatment outcomes.

## INTRODUCTION

The opioid crisis has taken an unprecedented global toll. Worldwide, in 2020, approximately 61 million people reported past‐year non‐medical opioid use (a twofold increase since 2010). Although it is estimated that opioids account for 35% of substance use disorders (SUD), they account for 77% of drug‐related deaths [1]. In the United States (US), 79 770 overdose deaths involved opioids in 2022 [2], with toxic unregulated drug supplies of synthetic opioids (such as fentanyl) and stimulants driving mortality [3].

To combat the opioid crisis, evidence‐based treatments with medications for opioid use disorder (MOUD), including methadone, a full opioid agonist, and buprenorphine, a partial opioid agonist that can be combined with naloxone (an opioid antagonist used to reverse opioid overdoses) under the brand name Suboxone, have been recommended by the World Health Organization [4]. However, despite the strong and ever‐growing evidence of the effectiveness of MOUD in saving lives [4], reducing overdose risk [5, 6] and improving quality of life among people with opioid use disorder (POUD), treatment access, uptake and retention have remained low in the United States [7]. It is estimated that only 22% of POUD received MOUD in 2021, and the average duration of MOUD was under 6 months, which is insufficient to reduce mortality at the population level [8].

Achieving longer retention and supporting medically supervised treatment are paramount for making MOUD treatment safe and effective. Adopting a person‐ and patient‐centered approach is increasingly recognized as a path forward to better treatment outcomes. Understanding and integrating medication experiences into healthcare provision has the potential to address previously under‐appreciated challenges faced by patients, leading to better adherence and retention in treatment [9].

The field of medication experience is growing, with relevant frameworks describing both medications' physiological and psychological effects [9, 10, 11, 12, 13, 14]. As such, medications' bodily effects, the symbolic meanings attributed to medications and patients' previous experiences, expectations and concerns, current life circumstances, feelings and social pressures influence uptake, adherence and retention [14]. A recent systematic review of qualitative studies investigating MOUD in the United States identified patients' lived experiences as one of the key themes influencing MOUD trajectories. These included logistical barriers and costs, secondary effects of the medication, intentional non‐adherence for diversion or sporadic use and lack of social support, including from healthcare providers because of frequent dissonance between providers' and patients' treatment goals [15]. However, the authors acknowledged that one of the study's limitations was excluding studies using data from social media.

Social media can provide rich data on MOUD trajectories, offering virtual spaces to communicate, socialize and share experiences. In particular, Reddit has become one of the most used social media platforms in the United States [16]. Reddit's uniqueness relies on its thematic forums and participants' anonymity, fostering in‐depth engagement less prone to social desirability bias. Reddit exploration offers valuable insights into general health‐related issues [17], substance use [18] and, more specifically, opioid use experiences [19].

We aimed to contribute to the field of medication experiences among POUD by analyzing Reddit data from the r/Methadone and r/suboxone subreddits, two discussion rooms dedicated to experiences with methadone and buprenorphine‐related medication. We used natural language processing (NLP) methods to unveil experiences related to each medication type. Our objectives were to (i) identify and describe the main topics discussed in these two subreddits relevant to MOUD use/retention; (ii) characterize differences in experiences between the two medications; and (iii) discuss appropriate strategies to overcome the challenges faced by those receiving MOUD.

## METHODS

### Data source

We used Reddit as the data source for the analysis. Reddit is organized in moderated thematic forums (subreddits) where people can post questions and answers or share experiences. It had around 82.7 million daily active Redditors (i.e. users/members) in 2024 [16].

We explored the two subreddits on MOUD with the largest number of members: r/Methadone and r/suboxone, representing over 75% of members participating in MOUD‐focused subreddits (Appendix A). We analyzed all titles and initiating submissions (called ‘posts’ hereafter) from the subreddits' creation in 2011 until 31 December 2022. We excluded polls and repeated submissions. Comments were not included to prevent topic drift [20].

### Data analysis

We used NLP methods for this analysis as they allow the processing of extensive collections of posts (i.e. ‘text corpus’) by extracting specific data from them and offering statistical tools to draw inferences about a research topic. We used a three‐stage process for the NLP analysis: (i) prepare and characterize the corpus (Appendix B.1); (ii) estimate independent topic models for r/Methadone and r/suboxone; and (iii) label, validate and group the topics.

We analyzed 37 278 first posts and titles from r/Methadone (14 828) and r/suboxone (22 450) subreddits (Appendix C presents the subreddits descriptive analysis). To explore differences between the subreddits, we used mutual information, a statistic that quantifies how much a term contributes to classify a post as belonging to r/Methadone or r/suboxone, and unigram, bigram and trigrams word clouds (Appendix D).

We, then, estimated a correlated topic model (CTM) proposed by Blei and Lafferty [21] for each subreddit to unveil the main topics discussed by Redditors. This unsupervised method assumes topics may correlate and estimates posts' share (i.e. the percentage of each post attributed to each topic) in all emerging topics. As the number of topics is a critical step to determine the more appropriate CTM solution, we relied on substance use experts' (A.A. and A.B.) iterative evaluations of the solutions and metrics such as semantic coherence, held‐out likelihood, exclusivity and residual [22].

**FIGURE 1:**
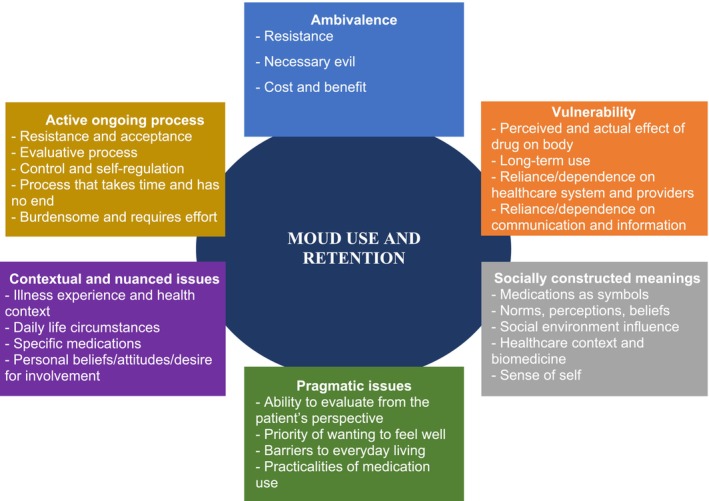
Diagram of the medication experience framework's attributes and their main elements potentially influencing medications for opioid use disorder (MOUD) use and retention.

We labeled topics according to their meaning, through interpreting their more exclusive and prevalent words and deeply reading each topic's 10 most representative posts. We, then, estimated each topic's average topic share (TS) to infer its importance/frequency. We leveraged the Hillman *et al*. [9] medication experience framework to characterize each topic. This framework describes the individuals' subjective experiences of taking medications into six key attributes: ambivalence, vulnerability, socially constructed meanings, pragmatic issues, contextual issues and the use of medications as an active ongoing process (Figure 1) [9]. We used inter‐topic correlations and substance use analysts' interpretation to manually group the topics into macro‐meso classes and the medication experience framework attributes (Appendix B.2). Within each macro‐class, topics were grouped into meso‐classes based on similarities. We illustrated the topics by providing synthetic quotes, following Benton *et al*. [23] ethical suggestions (Appendix B.3).

We described and compared r/Methadone and r/suboxone topics relevant to MOUD use and retention challenges within each macro‐ and meso‐class. When referring to topics from each subreddit, we specified ‘M’ and ‘S’ initials alongside the topic number (e.g. ‘M10’ for r/Methadone topic 10). We provided the topic share and name in the tables' first columns. For readability, we only presented topics with average topic shares totaling more than 5% (considering r/Methadone and r/suboxone) and presented the remaining topics in Appendix H.

The University of California, San Diego institutional review board waived this study as not human subject research (805709). The software used is described in Appendix B.4.

## RESULTS

**FIGURE 2:**
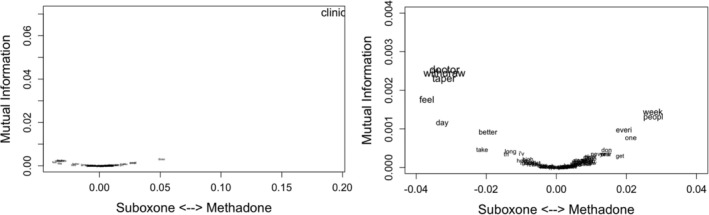
Mutual information showing terms that better differentiate the subreddits. Left, no zoom, showing the impact of ‘clinic’ in r/Methadone. Right, zoomed picture to understand the subreddits secondary differences.

Based on the mutual information exploration, we found that r/Methadone's discussions differed from those in r/suboxone mainly because of Redditors' experiences with ‘clinic’ and, zooming in, the most distinctive discussions in r/suboxone were their experiences with ‘doctor’, ‘taperin’ and ‘withdrawal’ (Figure 2).

**FIGURE 3:**
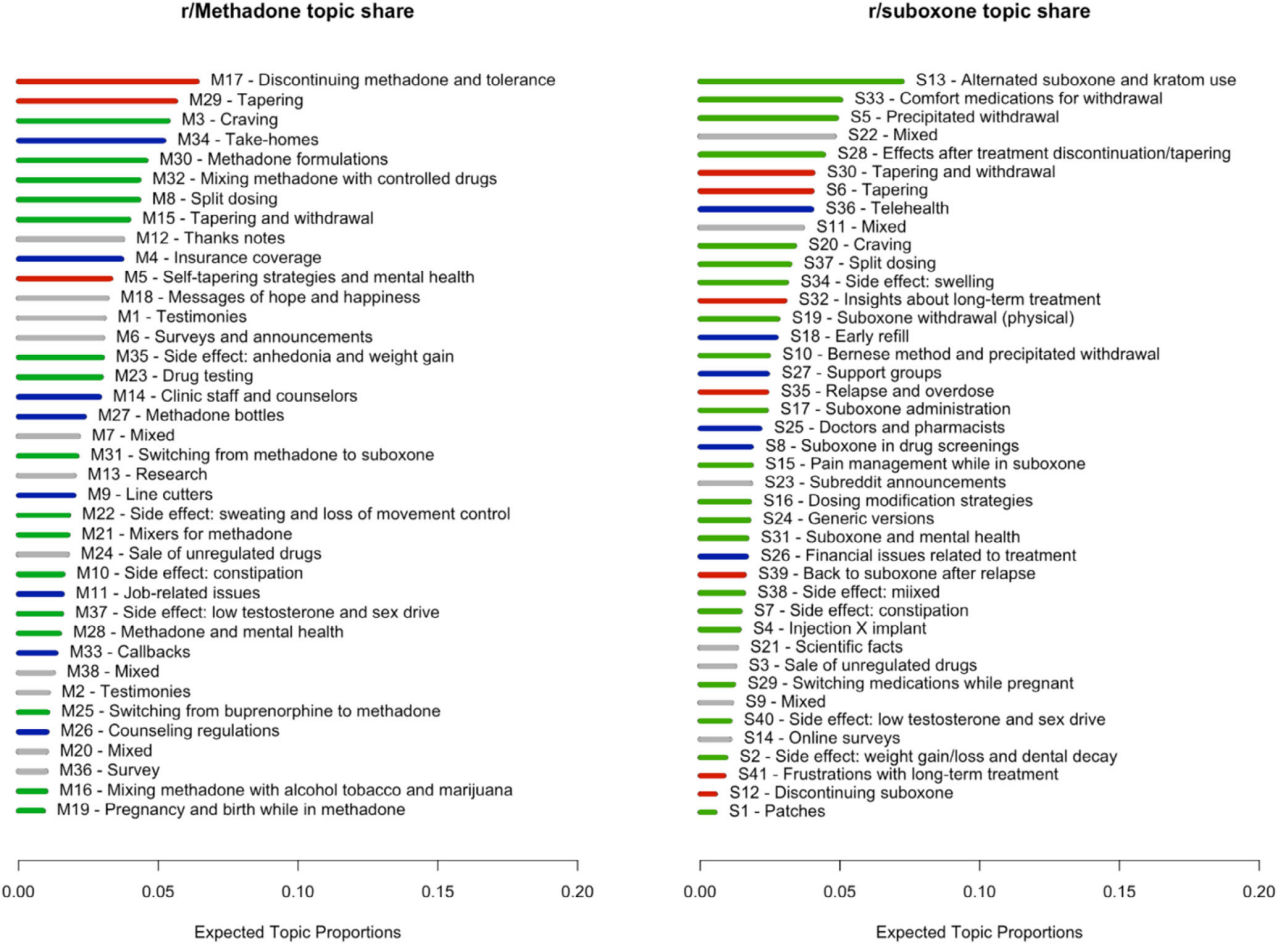
Average topic share by subreddit. Blue bars denote topics related to ‘healthcare‐related issues’, green bars denote ‘medication‐related issues’ topics, red bars denote ‘treatment discontinuation’ topics, and gray bars are topics with mixed themes or not related to medications for opioid use disorder (MOUD) treatment challenges, and therefore, not analyzed in this study.

**FIGURE 4:**
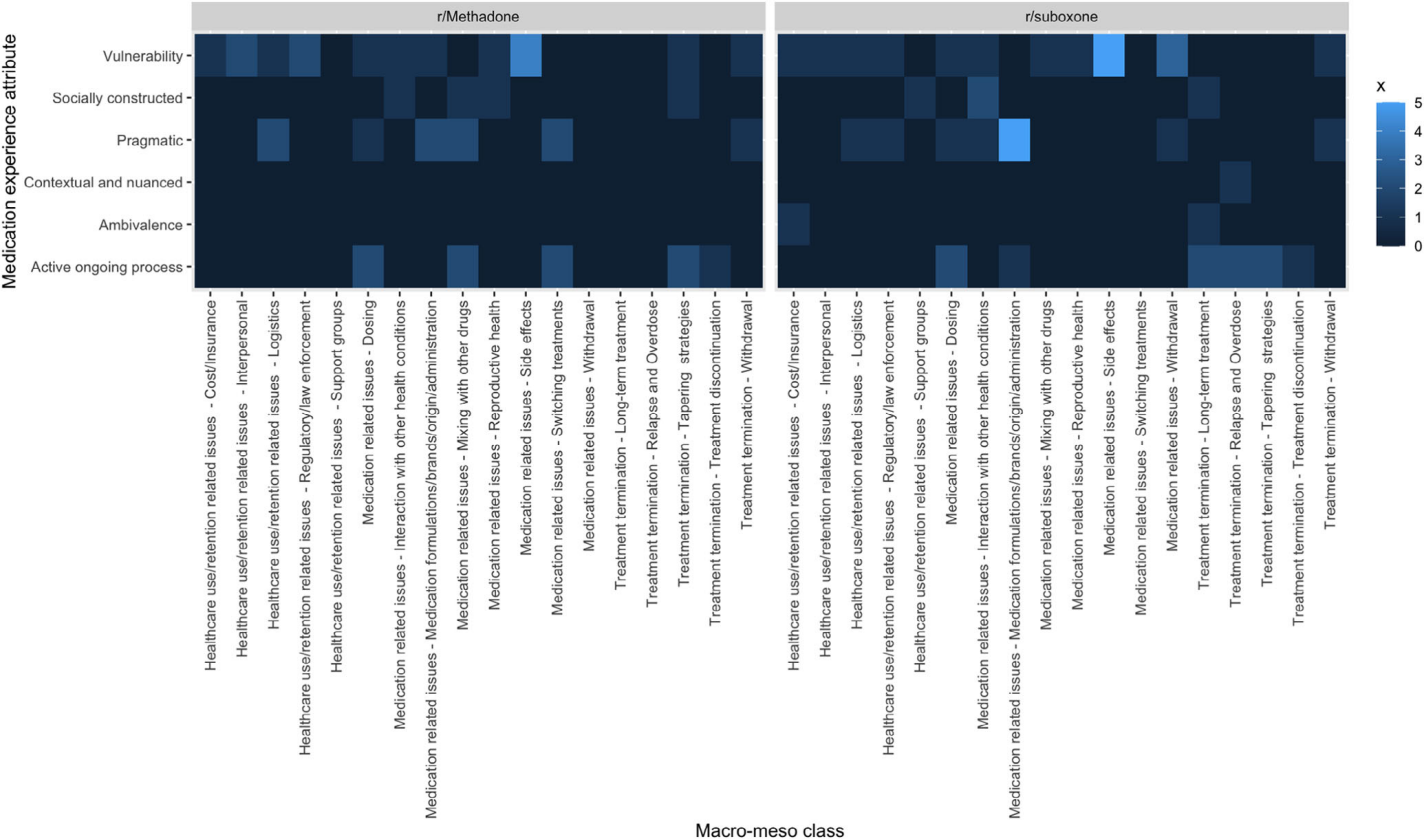
Number of topics by macro‐meso class and medication experience framework's attribute.

The final CTMs had 38 and 41 topics for r/Methadone and r/suboxone, respectively (sensitivity analysis in Appendix E). Based on the inter‐topic correlation (Appendix F) and analysts' expertise, we identified three macro classes: (i) healthcare‐related issues; (ii) medication‐related issues; and (iii) treatment discontinuation. Other topics not relevant to this analysis are presented in Appendix G. For each subreddit, the average topic share by macro‐meso classes is presented in Table 1, and the individual topic shares in Figure 3. The number of topics by macro‐meso class and by medication experience framework's attribute is illustrated in Figure 4.

**TABLE 1:** Topic share by macro‐meso classes of challenges of MOUD use and retention.

	r/Methadone (%)	r/Suboxone (%)
Healthcare‐related issues	20.05	14.74
Logistics	8.90	6.71
Interpersonal	4.88	2.14
Support groups	–	2.41
Cost/insurance	3.69	1.66
Regulatory/law enforcement	2.58	1.83
Medication‐related issues	41.55	53.87
Medication formulations/brands/origin/administration	6.33	7.80
Switching treatments	3.13	–
Dosing	9.65	6.58
Withdrawal	3.94	19.53
Mixing with other drugs	8.24	7.22
Side effects	7.92	8.05
Interaction with other health conditions	1.46	3.50
Reproductive health	0.88	1.19
Treatment discontinuation	15.31	16.43
Tapering strategies	8.92	8.04
Long‐term treatment	–	3.88
Treatment discontinuation	6.39	0.55
Relapse and overdose	–	3.96
Other	23.09	14.96

Abbreviation: MOUD, medications for opioid use disorder.

### Healthcare‐related issues

#### Logistics

Logistical issues highlight pragmatic and vulnerable aspects of the medication experience for people on MOUD because they involve everyday practicalities of medication use and reliance on the health system, respectively. Logistical challenges were markedly different for people in methadone and suboxone. People in r/Methadone reported problems with (i) transporting and preserving the medication (M27); (ii) getting take‐homes during holidays/weekends (M34); and (iii) callbacks (clinics requirement to inspect patients' methadone take‐homes, M33). For people in r/suboxone, logistical issues were less common and related to early refill timing and regulations (S18) and telehealth access issues, such as webpage navigation, login and phone contact (S36).

**Table:** 

Topic	Excerpt	The medication experience attribute
M27 – Methadone bottles (TS = 2.35%)	‘The last time I flew with methadone, I carried it with me very carefully […]. Well, when I arrived at my destination and checked the lockbox, it looked like the bottle had exploded.’	Pragmatic
M34 – Take‐homes (TS = 5.20%)	‘[…] Can I take Sunday and Monday's Labor Day as take‐home, or can I only take Monday's Labor Day?’	Pragmatic
M33 – Callbacks (TS = 1.34%)	‘How are bottle checks performed at the [X facility]? I am asking because I have been told that I missed a bottle check. I have two phone numbers, a contact email address, and permission to leave messages. I went today to see I was downed for failing the bottle check on 12/15 and now I have lost my take‐home and I am back on daily dosing.’	Vulnerability
S18 – Early refill (TS = 2.72%)	‘Early refills are usually talked about in the context of monthly scripts, but I was curious about biweekly scripts […]. Does the early refil rule apply to biweekly schedule scripts like this?’	Pragmatic
S36 – Telehealth (TS = 3.99%)	‘What's going on with Quickmd? I was scheduled to have a medical checkup yesterday, but the medical provider canceled my appointment. I'm getting an error when trying to log in today. I tried calling, but the number has been disconnected?’	Vulnerability

#### Interpersonal

People on both medications expressed vulnerability as they depend on health staff and the system's organization to obtain treatment such as adverse interactions with clinic staff, counselors and peers (such as dealing with ‘line cutters’), missing appointments, incorrect information on files and difficulty obtaining drug test results (M9 and M14) in r/Methadone, and doctors and pharmacy staff misconduct, stigma or indiscretion (S25) in r/suboxone.

**Table:** 

Topic	Excerpt	The medication experience attribute
M9 – Line cutters (TS = 1.98%)	‘I do not understand why people get up and try to open the door when there are 4, 5, 10, 11 people lined up in front of the door? […]’	Vulnerability
M14 – Clinic staff and counsellors (TS = 2.90%)	‘My counselor called me a second time for an appointment to do a bio‐psych and finish the intake. I rescheduled, but she was absent again. I rescheduled it again for 6 am yesterday. I showed up, but again she wasn't there. She tried to say that I changed my plans and missed the appointment, which is a complete lie and she is the one missing work and our appointment.’	Vulnerability
S25 – Doctors and pharmacists (TS = 2.14%)	‘I was talking to my sister today and she told me that she is looking for a new doctor because he made her take off all of her clothes during her appointment because “she needed to be tested for an abscess”! Hmm…what? I told her I would have left right away! I also told her to report him […]. As she took off her clothes he seemed to get even creepier and even touched her! If he's looking for an abscess, why touch her? […]’	Vulnerability

#### Cost/insurance

Treatment cost was a common issue in both subreddits. It reflected people's vulnerability in a complex health system in which information about insurance coverage is unclear, and patients often have to contribute financially. Although a topic about (public and private) insurance coverage emerged in r/Methadone (M4) and in r/suboxone, Redditors discussed treatment prices (S26), often comparing them with the cost of non‐prescribed opioids, highlighting their financial ambivalence.

**Table:** 

Topic	Excerpt	The medication experience attribute
M4 – Insurance coverage (TS = 3.69%)	‘Does anyone know if insurance covers methadone clinics in North Carolina? I have Blue Cross Blue Shield […]’	Vulnerability
S26 – Financial issues related to treatment (TS = 1.66%)	‘How on earth can you afford to go to a suboxone clinic and buy suboxone? […] I found several suboxone clinics nearby and called them all. The average cost per visit is $200. From what I've researched, the total cost of monthly suboxone treatment at the clinic […] is pretty close to what I spend on oxycodone monthly.’	Vulnerability and ambivalence

### Medication‐related issues

#### Medication formulations/brands/origin/administration

Redditors showed concerns and insecurities regarding their medications. In particular, they lacked information on changing the medication formulation, brands or regimen. Pragmatic questions/advice on dealing with medication‐related discomforts, modifying their medication's administration or dosage, or confirming the perceived effects of different formulations were frequent. In some cases, these questions reflected vulnerability because of limited information and treatment challenges as part of an ongoing process. In r/Methadone, people discussed methadone formulations, interactions, ways to increase its bioavailability and to source the substance from the darknet to avoid the treatment/clinics' regulations (M30). Additionally, they also discussed mixers (i.e. soft drinks) to improve methadone's taste (M21). In r/suboxone, people questioned the differences between buprenorphine patches (S1), injection and implant options (S4), splitting pills (S16), administration (S17), as well as the difference between generic and branded versions of suboxone (S24).

**Table:** 

Topic	Excerpt	The medication experience attribute
M30 – Methadone formulations (TS = 4.56%)	‘Which formulation of methadone do you prefer to take? I am currently taking metadol‐D, and I prefer it over methadone […]’	Vulnerability and pragmatic
M21 – Mixers for methadone (TS = 1.77%)	‘Am I the only one who notices that the Mountain Dew perfectly balances out the bitter cherry taste from the dosage? […]’	Pragmatic
S1 – Patches (TS = 0.53%)	‘[…] What is the subutex dose equivalent to a 10 microgram per hour buprenorphine patch?’	Pragmatic
S4 – Injection X implant (TS = 1.40%)	‘I'm looking for experience with buprenorphine subcutaneous implant and injection […]. They both seem pretty expensive. Has anyone used them to taper? If so, how did they work?’	Pragmatic
S16 –Dosing modification strategies (TS = 1.76%)	‘[…] I keep seeing people asking about cutting down small strips, pills, asking how to get less than 1 mg etc. The answer is the volumetric dose and everyone tapering should be aware about it as it is the most effective way to get a dose that is quite small.’	Pragmatic and active ongoing process
S17 – Suboxone administration (TS = 2.37%)	‘Is […] Suboxone well absorbed under the tongue when it is pressed against the teeth and gums? Suboxone sometimes falls under my tongue and sticks to the gums in my mouth.’	Pragmatic
S24 – Generic versions (TS = 1.74%)	‘What are people's opinions and experiences with the Sandoz brand? I know it's a generic brand owned by the company that makes suboxone, […] but is there any difference? I've taken Mylan brand before oxycodone when I jam, and I thought it was weaker, but I wasn't sure […]’	Pragmatic

#### Dosing

Two distinct issues raised by Redditors related to the active ongoing process of finding their optimal dose: (i) craving related to likely underdosing and (ii) challenges associated with dose stability. In r/Methadone, the questions encompassed splitting doses and its relationship with mitigating side effects, tolerance after reaching a ‘stable dose’ and how long their starting dose kept them stable (M8). In r/suboxone, the dosing issues were also related to finding a ‘stable dose’, split dose timing and the administration of extra doses when one is missing (S37). These issues highlighted not only Redditors' attempt to exert control over their treatment as part of the active and ongoing medication process, but also the vulnerabilities related to their perception of the medication's effect and pragmatic solutions to achieve stability.

**Table:** 

Topic	Excerpt	The medication experience attribute
M3 – Craving (TS = 5.35%)	‘I have a strong desire to use it today. It's very difficult. I'm on methadone for 3 months now […]. I have a strong urge to use it, but I know I should not because taking it will set me back in phases. I'm on 60 ml and I still get cravings and the urge to get high, but I've heard that once you find a stable dose, the cravings should go away?’	Vulnerability and active ongoing process
M8 – Split dosing (TS = 4.30%)	‘Split dosing questions for those taking split doses: How long should I wait for my second dose of the day? What does your doctor recommend about dosing times? I am splitting doses and I still have problems with withdrawal, especially if I am very active or exercise. I wonder if changing the dosing time would help […]’	Pragmatic and active ongoing process
S20 – Craving (TS = 3.38%)	‘How well does suboxone suppress your craving? Even if I take very large doses (8 to 16 mg) per day, I still feel the urge to use from time to time.’	Vulnerability and active ongoing process
S37 – Split dosing (TS = 3.21%)	‘I take 12 mg of Suboxone twice a day. My morning dose is 8 mg and my second dose is 4 mg. I usually take them 4 hours apart. My question is: is this window too narrow? Will my first dose be canceled if I take the second too soon?’	Pragmatic and active ongoing process

#### Withdrawal

Withdrawal was a secondary subject of various topics in both subreddits. For r/Methadone, withdrawal emerged in topics describing mixing methadone with other drugs (M16 and M32), craving (M3) and tapering (M29), described in the appropriate sections. Specific symptoms of withdrawal for people tapering were seen in M15. For r/suboxone, we found topics discussing precipitated withdrawal (rapid and intense onset of withdrawal symptoms after administering a partial agonist, S5), the Bernese induction method to prevent precipitated withdrawal (S10) [24], physical (S19) and mental (S28) effects of treatment discontinuation/tapering and comfort medications for some withdrawal symptoms (S33). Although we cannot ascertain whether the psychological experiences entirely relate to withdrawal symptoms (S28), the physical symptoms mentioned in these posts are clearly referred to as withdrawal symptoms, both highlighting the perceived effects of the treatment (vulnerability).

**Table:** 

Topic	Excerpt	The medication experience attribute
M15 – Tapering and withdrawal (TS = 3.94%)	‘Day 1 of methadone withdrawal, some background: […] in treatment for more than 2 years. My tapering schedule is reducing 5 mg every 4.5–5 months if I feel completely stable on my new dose … My symptoms are sweating, restless legs, feel slow, heavy, anxious, anhedonia.’	Pragmatic and vulnerability
S5 – Precipitated withdrawal (TS = 4.88%)	‘Is it precipitated withdrawal symptom or did I take too much? I took 6 mg on Friday morning and 8 mg on Sunday night. I felt relief for about an hour and then kept throwing up all night and all day.’	Vulnerability
S10 – Bernese method and precipitated withdrawal (TS = 2.44%)	‘I did a lot of research but could not find the right dosage to use Bernese's method. Is there a way to induce it quickly?’	Pragmatic and vulnerability
S19 – Suboxone withdrawal (physical) (TS = 2.78%)	‘Are leg cramps and restless legs symptoms of suboxone withdrawal?’	Vulnerability
S28 – Effects after treatment discontinuation/tapering (mental, lack of energy, stomach issues, sleep problems) (TS = 4.41%)	‘Tomorrow it will make two weeks off of Suboxone, and I still feel tired all the time. The melatonin helps me sleep better, and I am working out and going for walks. But my brain is always like, ‘Let us watch Netflix instead’. I just do not have much motivation, but I'm not depressed.’	Vulnerability
S33 – Comfort medications for withdrawal (TS = 5.02%)	‘Today is my last dose of Suboxone (0.25 mg). Can anyone tell me what comfort medication helped with your withdrawal symptoms?’	Pragmatic

#### Mixing with other drugs

Many Redditors worried about mixing their MOUD with other prescribed and non‐prescribed drugs, as part of the active ongoing medication process. Within r/Methadone, M16 and M32 described pragmatic concerns about mixing methadone with alcohol, tobacco, marijuana and controlled drugs. M23 addressed false positive drug test results, how other drugs appear in treatment‐related screenings and their repercussions for the treatment, reflecting the socially constructed regulated nature of MOUD treatment. Within r/suboxone, people discussed the perceived effects of the drug in the body (vulnerability) when alternating suboxone and kratom use (S13), how kratom can help deal with suboxone withdrawal and how to return to suboxone treatment after using kratom.

**Table:** 

Topic	Excerpt	The medication experience attribute
M16 – Mixing methadone with alcohol, tobacco and marijuana (TS = 0.97%)	‘Are there any dangers I should be aware of when smoking weed or delta 8 products?’	Pragmatic and active ongoing process
M23 – Drug testing (TS = 2.96%)	‘Will dhma show up on a pre‐workout test as a false positive for amphetamines on a clinic drug test?’	Socially constructed
M32 – Mixing methadone with controlled drugs (TS = 4.31%)	‘If I use 160–240 mg of oxy or about 0.5 g of heroin per use, what should be my methadone dose?’	Pragmatic and active ongoing process
S13 – Alternated suboxone and kratom use (TS = 7.22%)	‘Will taking suboxone after taking kratom cause precipitated withdrawal? It's been almost 24 hours since my relapse so I cannot take suboxone yet, but I do have kratom […]’	Vulnerability

#### Side effects

Many topics demonstrated Redditors' vulnerabilities regarding treatments' side effects. Side effects common to both treatments were: constipation (M10 and S7), low testosterone and sex drive (M37 and S40), weight variation (M35 and S2) and sweating/swelling (S34 and M22). For r/suboxone, we also found one topic with mixed side effects, such as dysphoria, edema, mental blocks, adverse emotional effects, anemia and anger (S38).

**Table:** 

Topic	Excerpt	The medication experience attribute
M10 – Side effect: constipation (TS = 1.59%)	‘Any recommendation for constipation? I'm having such a hard time that I'm bleeding down there. Are stool softeners or laxatives appropriate?’	Vulnerability
M22 – Side effect: sweating and loss of movement control (TS = 1.79%)	‘Sweat when falling asleep: Does this happen to others? When I fall asleep for, I break out in a sweat.’	Vulnerability
M35 – Side effect: anhedonia and weight gain (TS = 3.00%)	‘Does anyone else have problems with weight gain after taking methadone? […] I've always been 120–125 lbs, but I've gained at least 20 lbs. I am not fat, but I am definitely thicker now.’	Vulnerability
M37 – Side effect: low testosterone and sex drive (TS = 1.54%)	‘Does methadone affect sex drive? My boyfriend is on methadone. As soon as he started using it, our sex life decreased significantly. He blames it on the medication.’	Vulnerability
S2 – Side effect: weight gain/loss and dental decay (TS = 0.92%)	‘Has anyone lost a lot of weight while taking suboxone? I lost my appetite and lost a lot of weight […]. I do not feel like eating and am extremely underweight. I am very worried.’	Vulnerability
S7 – Side effect: constipation (TS = 1.43%)	‘Does anyone have any good ideas for regulating bowel movements? Stool softeners, laxatives, dietary changes? […]’	Vulnerability
S34 – Side effect: swelling (TS = 3.09%)	‘Does anyone else wake up in the morning with a swollen face, especially around the eyes? […]’	Vulnerability
S38 – Side effect: mixed (TS = 1.55%)	‘Mental Blocks: Has anyone dealt with something like mental mind blocks, critical thinking, or on‐the‐go thinking in interviews?’	Vulnerability
S40 – Side effect: low testosterone and sex drive (TS = 1.07%)	‘I am taking 1 mg of suboxone daily and I am wondering if it is contributing to my low libido.’	Vulnerability

### Treatment discontinuation

#### Tapering strategies

Tapering strategies were among the top 10 most discussed topics in r/Methadone and r/suboxone and are also part of the active ongoing treatment process. In M5, people were interested in self‐tapering and medications for addressing mental side effects. Topics M29, S6 and S30 encompassed various tapering facets, such as kick‐off strategies, consequent withdrawal and the final stages when taking very low doses.

**Table:** 

Topic	Excerpt	The medication experience attribute
M5 – Self‐tapering strategies and mental health (TS = 3.30%)	‘I want to self‐taper because the clinic is making it literally impossible to get off. I am aking 135 and want to get down as soon as possible […]. I'm very bad at math, so any help is welcome.’	Vulnerability, active ongoing process and socially constructed
M29 – Tapering (TS = 5.62%)	‘I am steadily tapering off my medication and am reducing my dose by 5 mg per week. I started at 100 and slowly worked my way down to 45. I have spent a week feeling the last drop, so I decided to stay at 45 for another week […]’	Active ongoing process
S6 – Tapering (TS = 4.00%)	‘Is it possible to taper without withdrawal? I am ready to abort my MAT which lasted 6 months and I am ready to start tapering. I take 8 mg daily.’	Active ongoing process
S30 – Tapering and withdrawal (TS = 4.04%)	‘I have been on Suboxone for about 2 weeks and I am planning to quit in the next day or two. I took 8–6 mg a day for the first week. The second week I took 4 mg for a day or two, then 1 mg a day for the last 5 days. I'm going to lower it to 0.5 tomorrow and then maybe 0.25. Do you think I can have bad withdrawals?’	Active ongoing process

#### Treatment discontinuation

Discontinuing treatment was the most mentioned topic in r/Methadone. It is a common desire in both MOUD treatments and often the result of dealing with the side effects of long‐term treatment. M17 was related to discontinuing methadone and people developing tolerance after its prolonged use. S12 mainly included messages of hope from people who stopped suboxone treatment.

**Table:** 

Topic	Excerpt	The medication experience attribute
M17 – Discontinuing methadone and tolerance (TS = 6.39%)	‘How do I get off? Thankfully, I do not use heroin anymore. However, I have been on methadone on and off for the past 7 years. I am paying a lot of money for it, but the fact is that I want to get sober. I've tried to stay sober for almost 2 months, and I come back every time. I do not know what to do!’	Active ongoing process
S12 – Discontinuing suboxone (TS = 0.55%)	‘Finally Free from suboxone: Please ignore the horror stories. If I can do it, you can do it. […] I do not spend much time here anymore, but I've picked up a lot of good information from this room, so I thought it was finally time to give something back to you all sexy and sober people. ‘	Active ongoing process

## DISCUSSION

We used NLP to identify and compare the main topics discussed in the subreddits r/Methadone and r/suboxone. Reddit proved to be a rich source of information about medication experience with MOUD and its influence on use/retention. Although each of the large and diverse topics identified deserves in‐depth attention, we provided a roadmap of patients' experiences on MOUD for the research community.

Our first macro‐theme, healthcare‐related issues, mainly related to the pragmatic aspect of the medication experience [9], shaped by the requirements, organization, restrictions and human interactions at the healthcare services delivering these medications, leading to patient vulnerability. Common confusion around health insurance coverage confirmed the complicated and inconsistent insurance system across US companies, providers and geographical locations [15]. Logistics and interpersonal issues were more prevalent in r/Methadone, corroborating previous findings of clients' preference for the flexibility offered by the partial agonist treatment [25], in contrast with the unpractical everyday methadone clinic visits criticized as punitive [25].

The second macro‐theme identified, medication‐related issues, highlighted not only patients' pragmatic use of medications, but also their vulnerability associated with side effects and concerns on how to manage their mental and reproductive health in the context of a siloed health system [9]. Redditors active in these subreddits are highly informed about MOUD and keen to learn more about the medication to guide their decisions. This has been described as a desire to exert control over the medication experience as a way to recover some of the freedom and independence lost in the treatment process [14]. By definition, POUD are experienced in the use of drugs and have experimented with different doses and frequencies in the context of non‐prescribed drug use. It is, therefore, natural that they apply these same skills in the context of treatment. This knowledge of pharmacology through experience has been defined as ‘folk’ or ‘lay pharmacology’ [14]. However, such experimentation deviates from the expectation of medication adherence or compliance as determined by medical standards. This might explain why people rely on these subreddits to seek advice and share experiences. In particular, as shown by others [26], Reddit became a key source of support to deal with withdrawal and side effects during the coronavirus disease 2019 pandemic, as POUD faced drastic changes in their social networks and their OUD care.

Experiences of side effects were highly represented with a diverse list of manifestations and severity levels. This again forms part of the vulnerability of taking medications for chronic diseases and acts as a critical driver in the assessment of cost versus effectiveness of remaining on treatment (ambivalence) [9]. Despite previous qualitative studies indicating client preference for buprenorphine over methadone because of side effects [27], our analysis showed not only a similar prevalence of side effects discussions, but also overlaps in unwanted effects such as constipation, weight variation and low testosterone and sex drive [28].

Additionally, POUD have to deal with other co‐morbidities, including mental health issues, and questions about combining MOUD with medications for depression and anxiety reflected the siloed treatment of these conditions. Withdrawal was a driving medication‐related theme for a higher number of topics in r/suboxone, which can partly be explained by the more frequent use of suboxone for detox purposes as compared to methadone. That said, withdrawal was discussed as a critical subject across various other topics in r/Methadone, hiding the net prevalence of this issue.

The third core topic we identified was treatment discontinuation, which is closely related to the active ongoing process attribute of the medication experience [9]. Tapering strategies was a theme that emerged similarly from both subreddits, and multiple protocols were shared by peers to aid in this process. Many patients report doing this on their own, as opposed to under the guidance of a healthcare provider, and some express frustration with their clinic hampering this process. As mentioned, medications are ascribed meanings, including the loss of ‘normality’ and freedom, and this can be particularly strong in the context of treatment for SUD, because taking medications is so tightly associated with the socially constructed concept of dependence. Stopping the medication is, therefore, assimilated to ‘full recovery’ and, through this process, also comes the liberation from stigmatizing labels and beliefs, in addition to burdening side effects and logistical constraints [27].

Our findings indicate a need for policies adopting patient‐centered care models, tailored to individual experiences and needs [29]. At the provider level, enquiring about patients' treatment goals (short, long‐term use, abstinence) and establishing flexible dosing and tapering strategies could help align treatment with clients' expectations, as put forward in the Methadone Manifesto and by other experts with lived experience [30]. Similarly, open discussions about patients' experimentation with MOUD dosing and mixing with other drugs could lead to stronger provider/patient partnerships [29]. Pilot programs with regular medication reviews purposefully eliciting patients' experiences could encourage them to share, improve dosage, address symptoms, cravings and withdrawal, reduce polydrug use and optimize their overall health, considering their broader clinical context. At the institutional/clinic level, interventions delivering structured education programs for MOUD patients about medication options, side effects and tapering, potentially delivered through clinics or digital platforms, can tailor treatment better and reduce some of the heavy physical burden experienced [30]. Simplifying the appointment system to integrate visits into patients' routines or implementing low‐threshold care services could also lift barriers to uptake and retention [31]. Integrating peer navigators to support patients initiating treatment or facing challenges (in addition to social workers) could lead to more open disclosure of problems and the identification of appropriate solutions. At the health‐system level, additional strategies to enable take‐home doses for patients on methadone would also address many of the obstacles related to daily clinic visits, as found by others [30]. Similarly, lifting punitive restrictions around missed appointments and bottle checks would reduce stress associated with MOUD treatment. Better integration between SUD, mental health and primary care services should be a priority to provide more effective care for patients on MOUD. Finally, incorporating MOUD patients' expertise to improve MOUD protocols through leveraging social media platforms (such as done in this study) and through formal consultations with patients and relevant stakeholders, including harm reduction, housing, social welfare and emergency care service providers, would support the design of treatment delivery models that are tailored to the realities of people living with OUD.

### Limitations

The major limitation in studies using Reddit data is the anonymity of participants, leading to an absence of information on their OUD diagnosis or key socio‐demographics, including geographic location. This is an issue when examining pragmatic barriers to MOUD treatment, as insurance systems/regulations differ within and between countries. However, as per Reddit's general demographics, we expect most posts to come from the United States [16]. The lack of socio‐demographic information is likely much less problematic when investigating physical experiences of medications and attempts to taper and discontinue treatment. A second concern is Reddit members' representativeness of all patients on MOUD, because they are self‐selected based on their engagement with the treatment and their interest in obtaining or sharing information/support. Patients on mandated/involuntary treatment or coerced into treatment by structural factors [30] (e.g. toxic supply, the illegality of heroin and stigma) and individuals with no or limited internet access or literacy are likely under‐represented. Third, in terms of challenges shared, barriers to effective treatment at the structural/policy or community level (e.g. law enforcement interference, housing, area deprivation and stigma) were less represented than in other studies, likely because people tend to focus on their immediate experiences of physical, interpersonal or logistical issues [32]. Finally, the unsupervised NLP method used has the advantage of being data‐driven, therefore, topics emerge, unveiling patterns in the data. However, for this reason, the accuracy of findings is difficult to evaluate. As mitigation strategies, we thoroughly analyzed a set of possible topic model solutions through quantitative statistics and qualitative evaluation of the within‐topic homogeneity and between‐topic heterogeneity. A note on reflexivity is provided in Appendix I.

## CONCLUSIONS

We have provided a structured overview of issues addressed in the r/Methadone and r/suboxone subreddits, reflecting a detailed knowledge of the medications among patients and the need for more control over their effects, side effects and discontinuation. Acknowledging this expertise and establishing stronger patients' partnerships with the healthcare team and system might result in better treatment outcomes.

## AUTHOR CONTRIBUTIONS

**Alexandra Almeida:** Conceptualization (equal); data curation (lead); formal analysis (lead); investigation (equal); methodology (lead); validation (lead); visualization (lead); writing—original draft (lead); writing—review and editing (lead). **Mike Conway:** Formal analysis (supporting); investigation (supporting); methodology (supporting); supervision (supporting); validation (supporting); writing—review and editing (supporting). **David J. Grelotti:** Investigation (supporting); validation (supporting); writing—review and editing (supporting). **Amarnath Gupta:** Writing—review and editing (supporting). **David Frank:** Investigation (supporting); writing—review and editing (supporting). **Annick Bórquez:** Conceptualization (equal); formal analysis (equal); funding acquisition (lead); investigation (equal); project administration (lead); supervision (lead); validation (equal); visualization (supporting); writing—original draft (equal); writing—review and editing.

## DECLARATION OF INTERESTS

None.

## Supporting information


**Data S1.** Supplementary Information.

## Data Availability

The data that support the findings of this study are available on Reddit and obtained through Pushshift API.
