# Supplementary material for: Medication experiences in the treatment of opioid use disorders: Insights from Reddit
Source: Addiction. 2025 Mar 13;120(8):1610–22. doi: 10.1111/add.70022 (PMC12215240; doi:10.1111/add.70022)
Supplement: Supplementary file 1 — Data S1. Supplementary Information. [file ADD-120-1610-s001.docx]

# Supplementary Material

## Appendix A - Subreddits focused on MOUD

We systematically searched subreddits focusing on suboxone and methadone treatment (and analogous medications with different brand names) and selected the two major ones: r/suboxone and r/Methadone. Below, we list the main subreddits specifically focused on MOUD and their number of members as of 26th November 2024.

| **Subreddit** | **Members** |
| --- | --- |
| r/suboxone | 41, 000 |
| r/Methadone | 40,000 |
| r/sublocade | 6,900 |
| r/naltrexone | 6,300 |
| r/suboxonerecovery | 4,800 |
| r/Subutex | 3,500 |
| r/Vivitrol | 879 |
| r/humans_on_suboxone | 557 |
| r/SublocadeSuccess | 397 |
| r/SuboxoneTreatment | 395 |
| r/Methadone_AskNAnswer | 266 |
| r/allthingsnaltrexone | 204 |
| r/zubsolv | 186 |
| r/Brixadi | 119 |

## Appendix B – Reddit data extraction, processing, interpretation, and presentation

### Data preparation

We first characterized the corpus by exploring the most frequent words, including uni, bi, and trigrams (i.e., single and groups of two and three words, respectively) to gain preliminary insight into the data and identify misspellings, slang, and abbreviations related to opioids which we added to the list compiled by Almeida et al. (2024). Bi and trigrams with a prevalence lower than 5% were excluded from the analysis. Data preparation also included converting all text to lowercase, stemming (reducing words to their stem, in which, for example, "prescription" and "prescribed" become "prescrib"), and removing stopwords (as "the", "of", "a", etc.).

### Methodology for grouping the topics

AA and AB independently grouped the topics according to the macro and meso classes as well as the medication experience framework attributes. In the second stage, they met to compare the grouping results and discussed divergences until both reached an agreement.

### Procedure to synthetically modify the posts used as examples

The synthetic quotes were obtained by back translating the original messages (using Google translate) from English to Japanese and back to English to avoid participants' reverse identification. The messages were all revised for meaning by AA as the lack of punctuation and the use of slang can divert their meaning.

### Software and packages used

We used the Pushshift API and Python 3.10.2 to scrape the Reddit data.^a^ For all statistical analyses, we used R version 4.2.2.^b^ Several packages were used to manage the dataset, including the STM version 1.3.6 and stmCorrViz version 1.3.^c,d^

^a^ Baumgartner J, Zannettou S, Keegan B, Squire M, Blackburn J. The pushshift Reddit dataset. In: Proc Int AAAI Conf Web Soc Media, ICWSM [Internet]. AAAI Press; 2020. p. 830–9. Available from: https://www.scopus.com/inward/record.uri?eid=2-s2.0-85090240203&partnerID=40&md5=4dd06fee7c82711fb1eba34117418ee3

^b^ R Core Team. R: A Language and Environment for Statistical Computing [Internet]. 2019 [cited 2020 May 31]. Available from: https://www.R-project.org/

^c^ Coppola A, Roberts M, Stewart B, Tingley D. Package ‘stmCorrViz’: A Tool for Structural Topic Model Visualizations [Internet]. 2022. Available from: <https://cran.r-project.org/web/packages/stmCorrViz/stmCorrViz.pdf>

^d^ Roberts ME, Stewart BM, Tingley D. stm: An R Package for Structural Topic Models. J Stat Soft [Internet]. 2019 [cited 2020 Nov 19];91(2). Available from: http://www.jstatsoft.org/v91/i02/

## Appendix C - Subreddits descriptive analysis

These communities counted 15,804 active Redditors and shared about 4% of them. Figure I and II illustrate the subreddit posts and active Redditors over time and overall membership. From about 200 monthly posts and 150 active Redditors in each subreddit in January 2020, these subreddits reached a peak of over 400 and 700 monthly posts and 300 and 500 active Redditors for the methadone and suboxone subreddits, respectively, in December 2021 and decreased or stabilized after that.

| 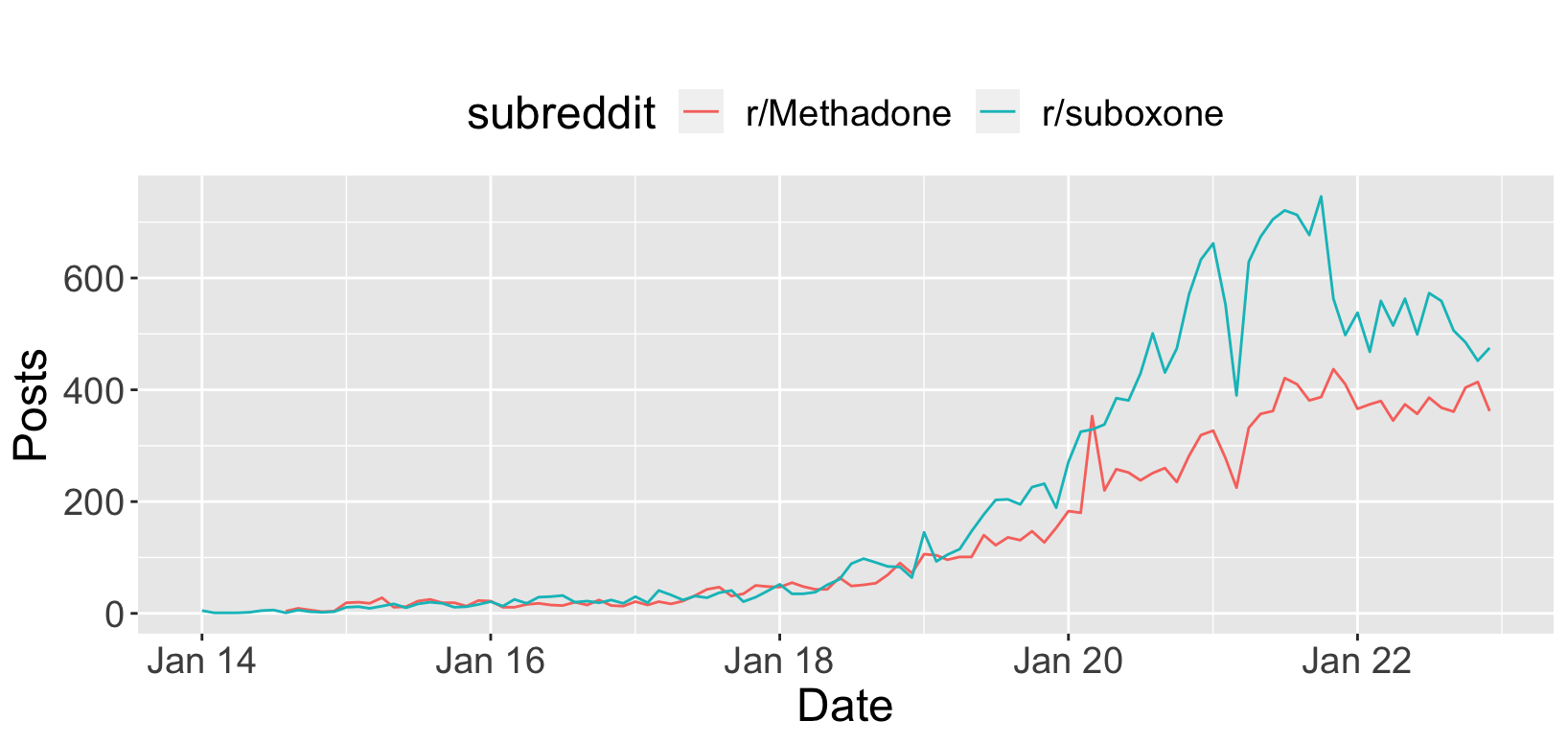 | 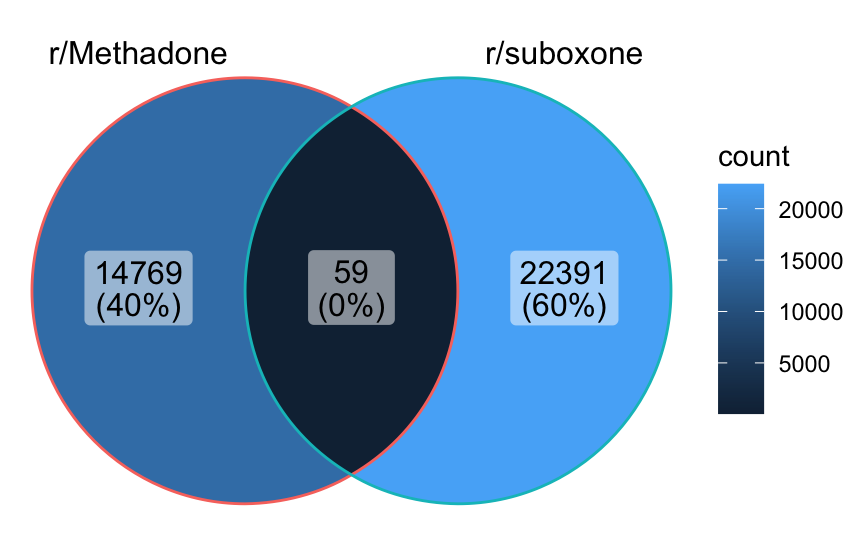 |
| --- | --- |

*Figure I - Monthly posts (left) and overall posts membership (right) by communities (r/Methadone and r/suboxone).*

| 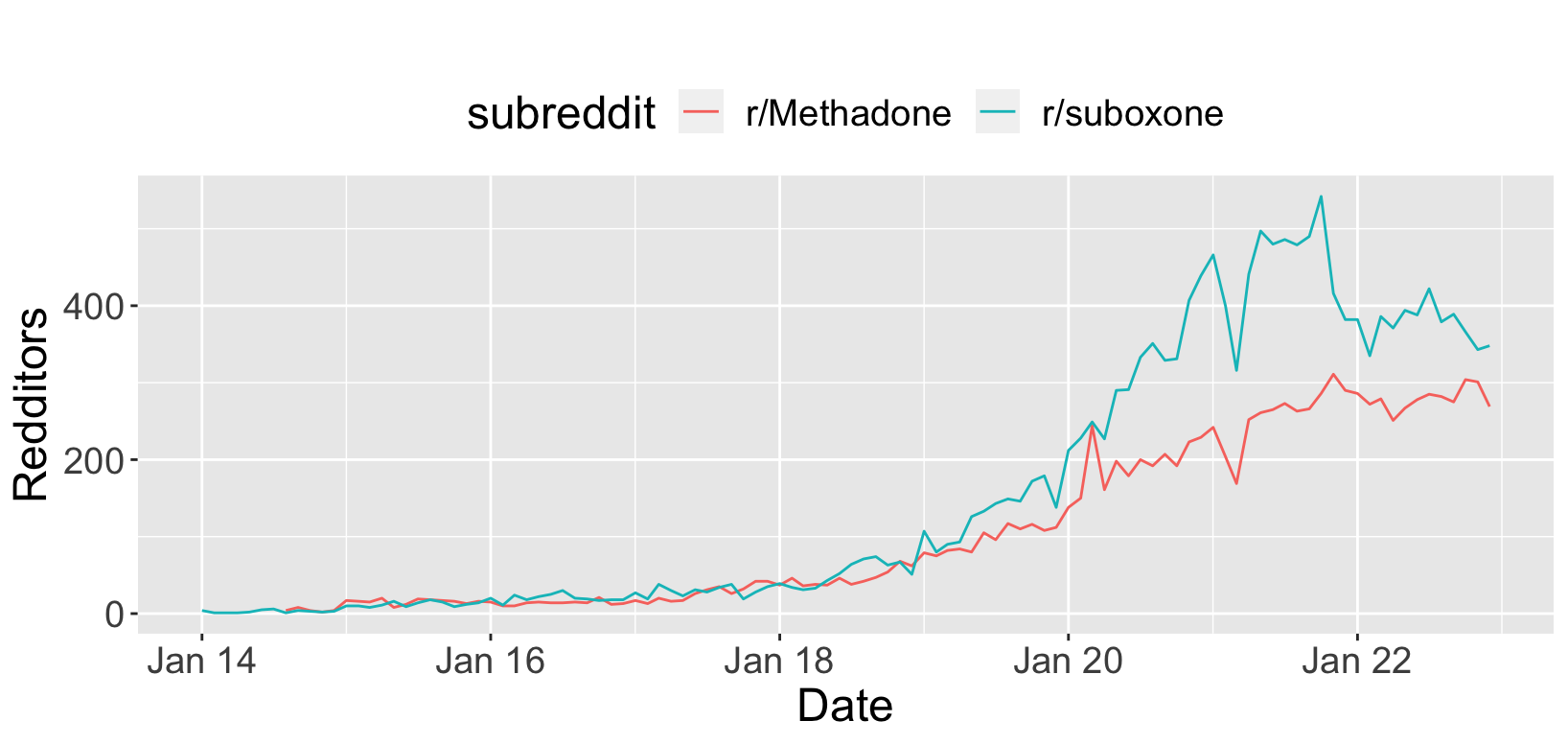 | 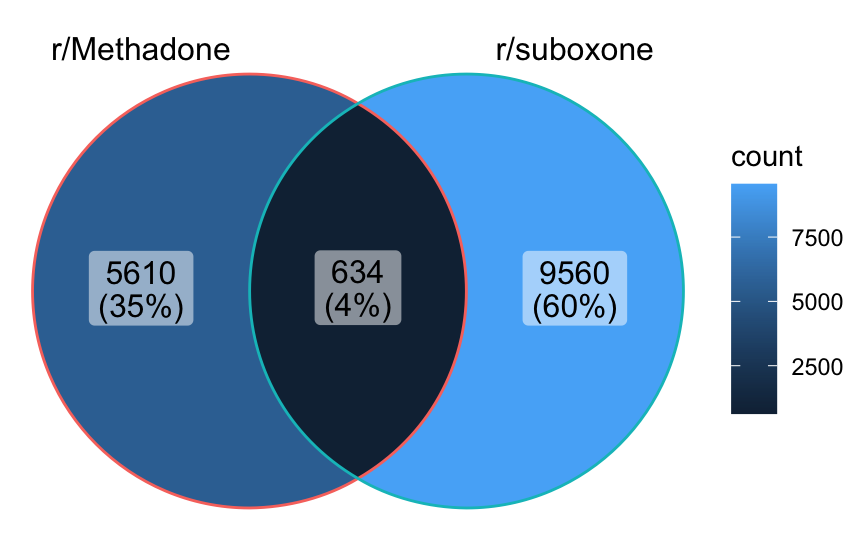 |
| --- | --- |

*Figure II - Monthly active Redditors (left) and overall active Redditors membership (right) by communities (r/Methadone and r/suboxone).*

## Appendix D – Words exploratory analysis: Mutual information mathematical definition; uni, bi, and trigrams world clouds with the most prevalent words in r/Methadone (1st line) and r/suboxone (2nd line). Terms with a prevalence lower than 5% were excluded.

## Mutual information formula:

##

$$\boldsymbol{M}\boldsymbol{I}_{\boldsymbol{kj}}\boldsymbol{=H}\left( \boldsymbol{k} \right)\boldsymbol{-H(k|j)}$$

## Where:

## k, referred as the “category”, is the subreddit from where the post is from.

##

$$\boldsymbol{H(k|j)=-}\sum_{\boldsymbol{k}\boldsymbol{,-}\boldsymbol{k}} \sum_{\boldsymbol{j}\boldsymbol{,-}\boldsymbol{j}} \boldsymbol{\pi}_{\boldsymbol{k}\boldsymbol{,}\boldsymbol{j}}\mathbf{log}_{\boldsymbol{2}} \boldsymbol{\pi}_{\boldsymbol{k|j}}$$

## $\sum_{\boldsymbol{k,-k}}$indicates the sum over posts in k and not in k

## $\boldsymbol{H}\left( \boldsymbol{k} \right)\boldsymbol{=-}\boldsymbol{\pi}_{\boldsymbol{k}}\boldsymbol{lo}\boldsymbol{g}_{\boldsymbol{2}}\boldsymbol{\pi}_{\boldsymbol{k}}\boldsymbol{-}\boldsymbol{\pi}_{\boldsymbol{-k}}\boldsymbol{lo}\boldsymbol{g}_{\boldsymbol{2}}\boldsymbol{\pi}_{\boldsymbol{-k}}$ is the entropy for category k

## $\boldsymbol{\pi}_{\boldsymbol{k}}$is the proportion of posts that fall into category k

## $\boldsymbol{\pi}_{\boldsymbol{-k}}\boldsymbol{=}\boldsymbol{1-\pi}_{\boldsymbol{k}}$ is the proportion of posts that don’t fall into category k

## $\boldsymbol{\pi}_{\boldsymbol{k,j}}$ is the proportion of posts that are both in category k and have the word j

## $\boldsymbol{\pi}_{\boldsymbol{k|j}}$ is the proportion of posts in category k, giving that the word j in present

## Source: Grimmer, J., Roberts, M. E., & Stewart, B. M. (2022). Text as data: A new framework for machine learning and the social sciences. Princeton University Press.

**Uni, bi, and trigrams world clouds**

| **r/Methadone unigrams** | **r/Methadone bigrams** |  |
| --- | --- | --- |
| 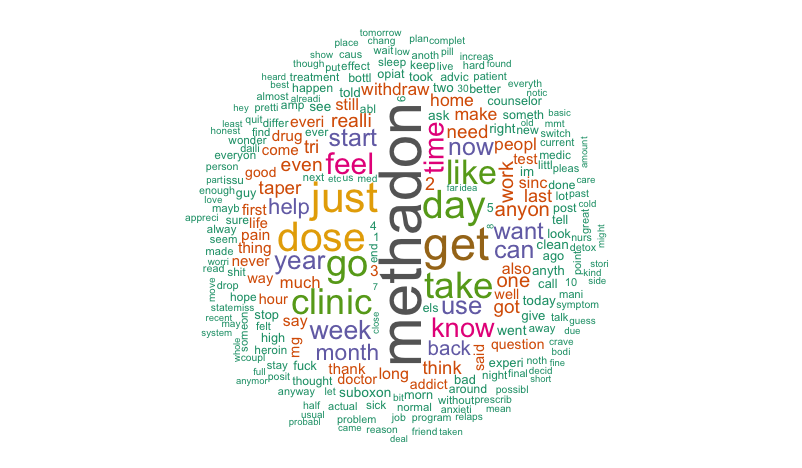 | 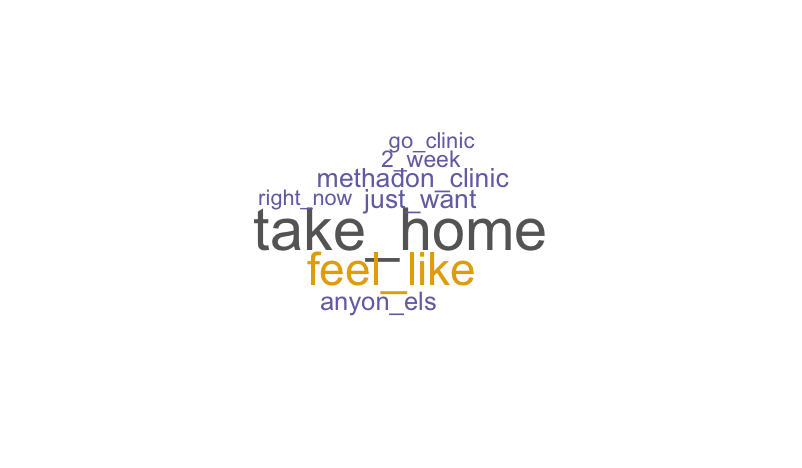 |  |
| **r/suboxone unigrams**  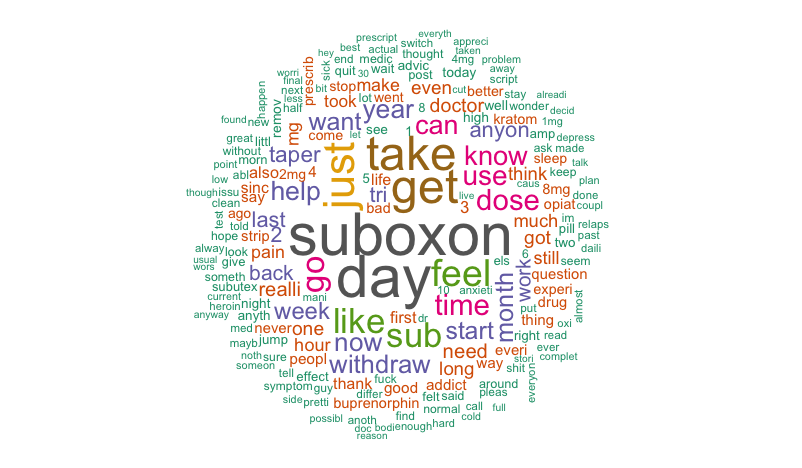 | **r/suboxone bigrams**  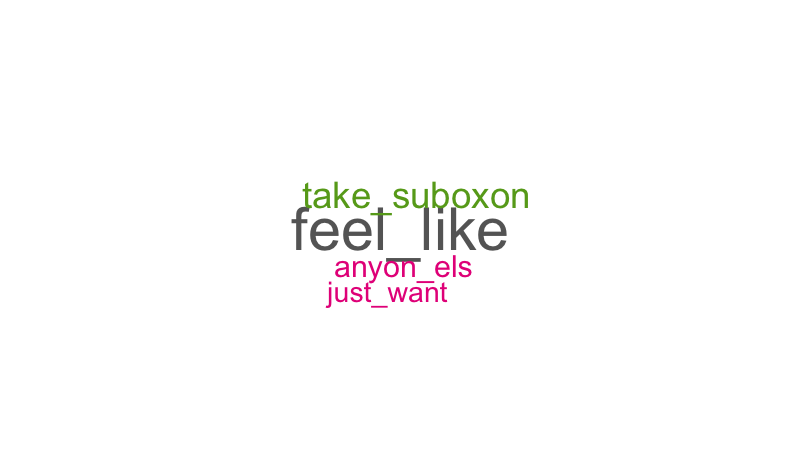 | **r/suboxone trigrams**  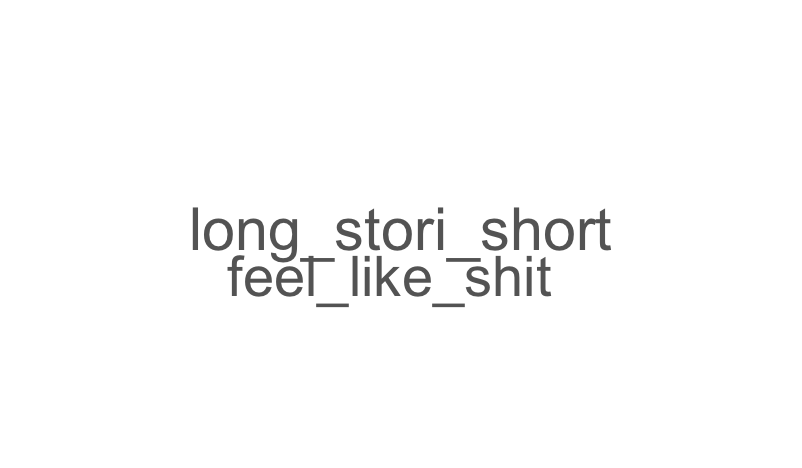 |

## Appendix E - Goodness of fit for r/Methadone and r/suboxone Correlated Topis Models with solutions ranging from 10 to 100

For both subreddits, solutions were examined based on coherence, exclusivity, held-out likelihood, and residual statistics. Based on these statistics, substance use researchers (AA and AB) analyzed the solutions for 7, 10, 16, and 20 topics (as used previously in the literature) and around 40-topic solutions (38, 39, 41, and 42 topics).

r/Methadone


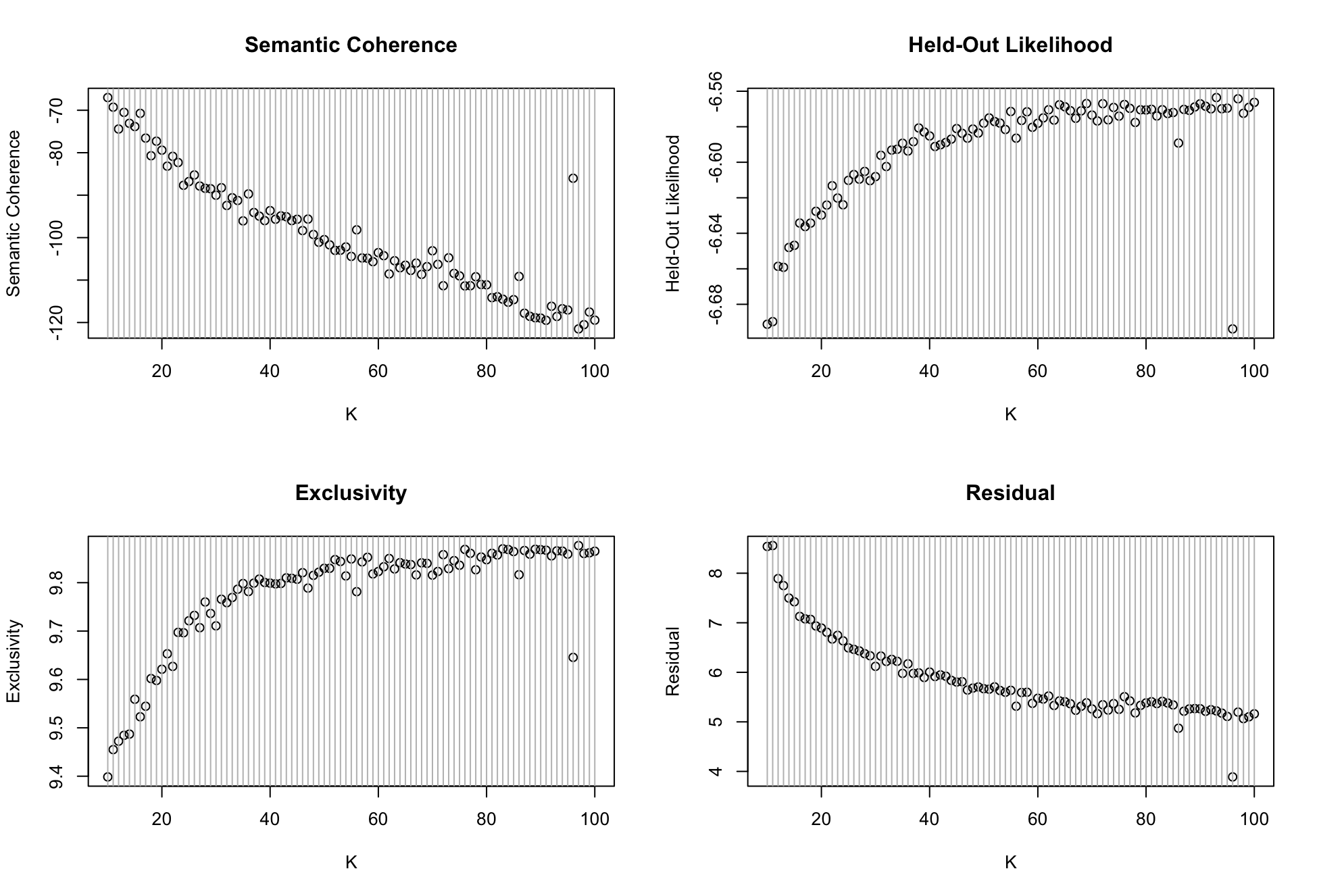


r/suboxone
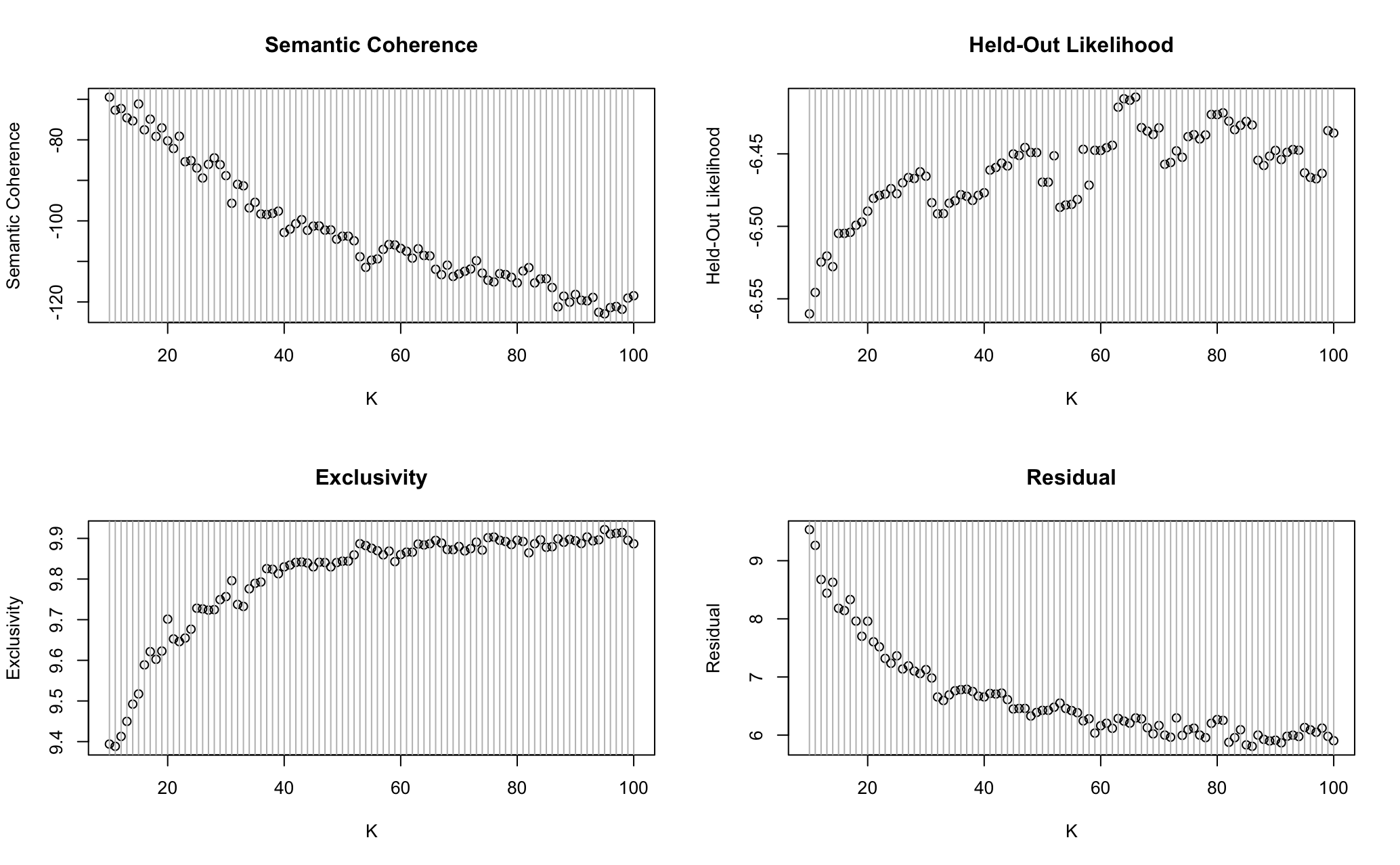


## Appendix F - Dendrogram based on the inter-topic correlation for r/Methadone 38-topic solution and r/suboxone 41-topic solution.

r/Methadone

r/suboxone

##

## Appendix G - Topics not analyzed in this study for bringing mixed or non-specific information to understand MOUD treatment use and retention

| r/Methadone | r/suboxone |
| --- | --- |
| Topic M1 - Testimonies | Topic S3 - Sale of unregulated drugs |
| Topic M2 - Testimonies | Topic S9 - Mixed |
| Topic M6 - Surveys and announcements | Topic S11 - Mixed |
| Topic M7 - Mixed | Topic S14 - Online surveys |
| Topic M12 - Thanks notes | Topic S21 - Scientific facts |
| Topic M13 - Research | Topic S22 - Mixed |
| Topic M18 - Messages of hope and happiness | Topic S23 - Subreddit announcements |
| Topic M20 - Mixed |  |
| Topic M24 - Sale of unregulated drugs |  |
| Topic M36 - Survey |  |
| Topic M38 - Mixed |  |

## Appendix H - Description and comparison of topics with average topic share lower than 5%

### Healthcare-related issues

#### Support groups

One of the few facilitators identified in Reddit for people in MOUD was the possibility of engaging in online support groups. This reflects the influence of the *socially constructed environment* on patients’ treatment experience, as the connection with others going through similar challenges helps manage them. This topic only emerged in r/suboxone (S27). It included the advertisement of support groups, people’s accounts of their experiences in them, and the recognition of r/suboxone as a community supporting people who need help.

| **Topic** | **Excerpt** | **The Medication Experience attribute** |
| --- | --- | --- |
| S27 – Support groups  (TS = 2·41%) | *"I just love our attitude with each other overall. This community [r/suboxone] really tries to push each other to live happily. It doesn’t matter if you are new to recovery or an old vet like me trying to have a decent life, we push success and happiness to everyone. I know that I personally would feel self-absorbed without this great community. thanks to you all"* | Socially constructed |

#### Regulatory/law enforcement

National and state-wide regulations around MOUD treatment coverage and job-related drug tests generate *vulnerability* among patients, as they impose conditions on their treatment or employment, which are often difficult to comply with, generating *pragmatic* questions and issues. Specifically, both subreddits unveiled job-related concerns. People in r/Methadone worried about losing insurance or methadone prescriptions between jobs when trying to get a job at a company with a pre-employment drug test or starting to receive salaries over the limit covered by public health insurance (M11). For people in r/suboxone, the main concern was related to suboxone appearing in job-related drug tests (S8).

| **Topic** | **Excerpt** | **The Medication Experience attribute** |
| --- | --- | --- |
| M11 – Job-related issues  (TS = 1·57%) | *"It's official: I lost my insurance because I made money again. Medicaid folks said: "In Florida, we give Medicaid to women and children! You now earn $851 a month on the books. $851 x 12 = limit exceeded". How can you average the last three salaries and turn it into a one-year forecast? Many people get frustrated. What happens if I lose this job in two months?"* | Vulnerability |
| M26 – Counseling regulations  (TS = 1·01%) | *"Does your clinic require group counseling sessions? I would like to know if other clinics are as strict as mine. I'm in Pennsylvania, and some treatment phases require group meetings. The lowest level is 6 hours per month. The minimum is 2.5 hours per month. There are approximately 5 to 6 one-hour groups each day. There is also a monthly individual counseling session required. All groups meet at 6 a.m. to 11 a.m. It's in the morning, so that's not possible for a lot of people who have jobs. They never offered evening groups."* | Vulnerability |
| S8 – Suboxone in drug screenings  (TS = 1·83%) | *"Does suboxone show up in city pre-employment test? Do they do the testing for government jobs like police or other city jobs? The policy and regulations document states the drugs tested. There's a summary of drugs listed. Is this accurate or can they hide tests for a drug not on the list?"* | Vulnerability and Pragmatic |

### Medication-related issues

#### Switching treatments

Issues related to switching medications (methadone from suboxone and vice versa) were found in r/Methadone only. They highlighted *pragmatic* considerations in the context of the *active ongoing treatment* *process* with MOUD. While the discussion on switching from buprenorphine to methadone focused on dose equivalences (M25), the switch from methadone to suboxone pointed out the difficulties of leaving a full agonist to start a partial agonist and its withdrawal effects (M31).

| **Topic** | **Excerpt** | **The Medication Experience attribute** |
| --- | --- | --- |
| M25 – Switching from buprenorphine to methadone  (TS = 1·04%) | *"Is there a way to calculate the equivalent methadone dose from buprenorphine? - I am considering switching from buprenorphine 6mg/day to methadone. I've been on 16mg for about a year, but have reduced it to 6mg in the last few months. Is there a way to calculate the amount of methadone equivalent to 6 mg of buprenorphine per day?"* | Pragmatic and Active ongoing process |
| M31 – Switching from methadone to suboxone  (TS = 2·09%) | *"Does methadone have a waiting period like Suboxone? I'm considering taking methadone for a short period of time just to detox, but is it like suboxone, where we must wait days to not be in precipitated withdrawal?"* | Pragmatic and Active ongoing process |

#### Interaction with other health conditions

The core concern in these topics covers the *socially constructed* biomedicine and health system context of managing MOUD treatment and other health issues. Redditors’ fear of having practitioners/pharmacies and doctors from other clinics accessing information about their MOUD treatment (through a database), because it may impact their mental health prescriptions (M28 and S31), reflects their *vulnerability* to depending on providers to prescribe these medications. In r/suboxone, users also questioned the effectiveness of pain medications when taking suboxone (S15) and reported how suboxone reduced or triggered their anxiety and risk of panic attacks (S31).

| **Topic** | **Excerpt** | **The Medication Experience attribute** |
| --- | --- | --- |
| M28 – Methadone and mental health  (TS = 1·46%) | *"Hey everyone, I see a psychiatrist and a methadone doctor at the clinic. My psychiatrist won't consider putting me on a benzo script, but I'm only staying with her because she's going to keep my addy script."* | Vulnerability and Socially constructed |
| S15 – Pain management while in suboxone  (TS = 1·83%) | *"Emergency Room: Severe pain due to a ruptured cyst. I spoke openly with my doctor about taking 2mg of Suboxone daily. He gave me an injection of Toradol to relieve the severe pain. Will this work?"* | Vulnerability and Socially constructed |
| S31 – Suboxone and mental health  (TS = 1·67%) | *"Suboxone is one of the few medications that has helped me with my anxiety and kept me from feeling depressed."* | Pragmatic and Socially constructed |

#### Reproductive health

The essential concern shared within these topics related to the *socially constructed* perceptions (stigma and social influence) of pregnant women in MOUD and reflected their *vulnerability* as key life outcomes for themselves and their child lies in the providers and health system’s values and regulations. M19 and S29 topics covered discussions on women’s reproductive health, such as changes in menstrual cycles, pregnancy, and birth-related issues while they are in treatment. In M19, women feared regulatory and staff procedures when giving birth due to limitations imposed by the treatment. Their concerns included the possibility of being separated from their baby after birth and law interferences. In S29, they also highlighted their insecurities in switching medications while pregnant.

| **Topic** | **Excerpt** | **The Medication Experience attribute** |
| --- | --- | --- |
| M19 – Pregnancy and birth while in methadone  (TS = 0·88%) | *"Since I'm about to give birth, I need all the advice and guidance I can get. First, please stop making insensitive comments about how selfish it is for women to take methadone during pregnancy. I'm not here for that. My due date is the first week of October, and I am currently on 40mg of methadone. I have some concerns regarding the involvement of CPS and babies born with NAS…”* | Vulnerability and Socially constructed |
| S29 – Switching medications while pregnant  (TS = 1·19%) | *"I'm 2 months pregnant and today my doctor switched me from Suboxone to Subutex. Moms, did it happen to you too?"* | Vulnerability |

### Treatment discontinuation

#### Long-term treatment

Many Redditors share perspectives about long-term treatment as an *active ongoing process*. Both topics identified were within r/suboxone. While S41 highlighted *ambivalence* towards long-term treatment and exposed complaints and side effects, S32 focused on specific questions such as why people taper after reaching 2mg, the side effects of suboxone long-term use, or motivation from people with a positive long-term experience.

| **Topic** | **Excerpt** | **The Medication Experience attribute** |
| --- | --- | --- |
| S32 – Insights about long-term treatment  (TS = 3·03%) | *"Does anyone know of any positive stories about long-term suboxone maintenance? I hear negative suboxone stories all the time from people who have been on suboxone for several years."* | Socially constructed and Active ongoing process |
| S41 – Frustrations with long-term treatment  (TS = 0·85%) | *"I’m tired of the suboxone “dance.” I've been on suboxone for 3 years now and it was a godsend for my opioid addiction, but I don't even think about that part anymore and now it's become a hassle in my daily life I cannot stop."* | Active ongoing process and Ambivalence |

#### Relapse and Overdose

Relapse was a theme partially explored in other topics, such as mixing methadone and suboxone with other drugs, tapering, and discontinuing treatment. Within r/suboxone, S35 described experiences of relapse and overdose among Redditors' acquaintances. S39 focused on reinitiating treatment after relapsing.

| **Topic** | **Excerpt** | **The Medication Experience attribute** |
| --- | --- | --- |
| S35 – Relapse and overdose  (TS = 2·38%) | *"I've been on Suboxone for a year and a half now (16mg a day). During this time, I've had various relapses...I've been on a month long 'bender' with 2 weeks between each use. All because I couldn't say goodbye to my old friend heroin."*  *“We lost another one to this epidemic. We are completely heartbroken. My neighbor lost his wife to an overdose in 2020. All his family live at the end of the driveway, but in separate houses. His mailbox was overflowing, and he wasn't seen for several days. The family asked for a welfare check. Heroin was discovered upon entry. He is gone! Another overdose…”* | Active ongoing process and Contextual and nuanced |
| S39 – Back to suboxone after relapse  (TS = 1·58%) | *"I dug myself into a big hole. I take over 1000mg a day. I take them orally and never sniff them. I can take 4 80s at oxycodone and still not feel it. To be honest, I don't even get high anymore. I'm thinking of stopping and starting suboxone. I tried to do this a few months ago and failed."* | Active ongoing process |

## Appendix I - Reflexivity

While an algorithm identifies topics through NLP, the topic solution selection, their labeling, aggregation, and interpretation are carried out by researchers and, therefore, influenced by epistemologies related to training and experience. In our case, as SUD epidemiologists, we were influenced by the philosophy of harm reduction and patient-centered approaches to care in the context of research. We purposefully included the perspective of a psychiatrist specialized in the treatment of SUD and a researcher with lived experience of treatment with MOUD to achieve a complete understanding of the experiences shared in these subreddits. Nevertheless, a different group of investigators would have likely emphasized the importance of other topics or interpreted them differently. A member-checking exercise in which these findings would be presented to members from these subreddits could shed light on this issue.
